# Rapid Quantification of Infectious *Cucumber green mottle mosaic virus* in Watermelon Tissues by PMA Coupled with RT-qPCR

**DOI:** 10.3390/v14092046

**Published:** 2022-09-15

**Authors:** Ali Chai, Quancheng Wang, Huajun Kang, Leiyan Yan, Yunping Huang, Yanxia Shi, Xuewen Xie, Lei Li, Tengfei Fan, Yuhong Wang, Baoju Li

**Affiliations:** 1 Institute of Vegetables and Flowers, Chinese Academy of Agricultural Sciences, Beijing 100081, China; 2 Ningbo Academy of Agricultural Sciences, Ningbo 315040, China

**Keywords:** *Cucumber green mottle mosaic virus*, propidium monoazide, RT-qPCR, detection, infectious, inactive

## Abstract

*Cucumber green mottle mosaic virus* (CGMMV) belongs to the *Tobamovirus* genus and is an important quarantine virus of cucurbit crops. Seedborne transmission is one of the principal modes for CGMMV spread, and effective early detection is helpful to prevent the occurrence of the disease. Quantitative real-time reverse-transcription PCR (RT-qPCR) is a sensitive and rapid method for detecting CGMMV nucleic acids, but it cannot distinguish between infectious and noninfectious viruses. In the present work, a propidium monoazide (PMA) assisted RT-qPCR method (PMA-RT-qPCR) was developed to rapidly distinguish infectious and inactive CGMMV. PMA is a photoactive dye that can selectively react with viral RNA released or inside inactive CGMMV virions but not viral RNA inside active virions. The formation of PMA-RNA conjugates prevents PCR amplification, leaving only infectious virions to be amplified. The primer pair cp3-1F/cp3-1R was designed based on the coat protein (*cp*) gene for specific amplification of CGMMV RNA by RT-qPCR. The detection limit of the RT-qPCR assay was 1.57 × 10^2^ copies·μL^−1^. PMA at 120 μmol·L^−1^ was suitable for the selective quantification of infectious CGMMV virions. Under optimal conditions, RT-qPCR detection of heat-inactivated CGMMV resulted in Ct value differences larger than 16 between PMA-treated and non-PMA-treated groups, while Ct differences less than 0.23 were observed in the detection of infectious CGMMV. For naturally contaminated watermelon leaf, fruit and seedlot samples, infectious CGMMV were quantified in 13 out of the 22 samples, with infestation levels of 10^2^~10^5^ copies·g^−1^. Application of this assay enabled the selective detection of infectious CGMMV and facilitated the monitoring of the viral pathogen in watermelon seeds and tissues, which could be useful for avoiding the potential risks of primary inoculum sources.

## 1. Introduction

*Cucumber green mottle mosaic virus* (CGMMV), a member of the *Tobamovirus* genus in the Virgaviridae family, is an economically important viral pathogen of cucurbit crops worldwide. It infects cucurbit species, such as watermelon (*Citrullus lanatus*), melon (*Cucumis melo*), cucumber (*C. sativus*), pumpkin (*Cucurbita moschata*) and bottle gourd (*Lagenaria siceraria*), causing serious losses to their production [1,2,3,4]. CGMMV was first described in cucumber plants in England in 1935 [5] and has subsequently rapidly spread to over 40 countries, including Greece, Bulgaria, Canada, the USA, Australia and Poland, achieving a global distribution [6,7,8,9,10,11,12,13]. In China, the occurrence of CGMMV in watermelon was first reported in 2006 and has now been found in Guangdong, Hunan, Jiangsu, Hainan, Zhejiang and Liaoning provinces and Ningxia Hui Autonomous Region. The most common symptoms of CGMMV-infected plants are leaf mosaic and mottling, growth stunting and malformation of leaves and fruits, which reduce the quantity and quality of fruit yield [5]. The global spread of CGMMV is mainly due to its highly stable viral particles, which are preserved in seeds, soil and plant debris [14,15,16]. CGMMV-infected seeds, seedlings or soil are the initial source of infection, followed by a massive secondary transmission that occurs during pruning or harvesting or through leaf contacts. In addition, CGMMV can also survive for a long time on diseased plants after infection [17]. Owing to its economic importance, CGMMV has been listed as a quarantine virus in many counties, including China [18]. Therefore, early and fast detection of CGMMV is of great significance for the prevention and control of the disease [19,20,21].

Commonly used methods, such as enzyme-linked immunosorbent assay (ELISA), reverse-transcription polymerase chain reaction (RT-PCR), reverse-transcription loop-mediated isothermal amplification (RT-LAMP), immunocapture-RT-PCR (IC-RT-PCR) and recombinase polymerase amplification assay (RPA) have been established and used for the qualitative detection of CGMMV [22,23,24,25,26,27,28]. However, it remains unclear whether PCR-positive samples are infectious. Recently, a quantitative real-time reverse-transcription PCR (RT-qPCR) assay was recommended for the quantitative detection of CGMMV due to its high sensitivity, reliability and specificity [29,30,31], but it could not distinguish infectious from noninfectious viruses effectively. The RNA from inactive virions can serve as a template in RT-qPCR amplification, resulting in the overestimation of infectious viruses in seeds or diseased plants. Therefore, there is still an unmet need for the rapid and convenient differentiation of infectious and inactive viruses for a better control of CGMMV transmission.

Propidium monoazide (PMA), an azide derivative, is a membrane-impermeable dye that is only able to enter damaged or destroyed capsids. Thus, the RNA inside live virions cannot be bound by PMA, and only the RNA from inactive virions can be bound by PMA. PMA has the property of being photoreactive and intercalating into RNA upon exposure to intense light, preventing their amplification by PCR [32,33]. It has been reported that PMA coupled with RT-qPCR can distinguish infectious viruses from inactive viruses of SARS-CoV-2, noroviruses (NoVs), adenoviruses (AdVs), rotaviruses (RoVs) and coronaviruses (CoVs) [34,35,36,37,38,39,40,41], which are important human and environmental viruses. However, few studies have combined PMA with RT-qPCR to differentiate infectious and inactive plant viruses, and none has been conducted on CGMMV.

The present study aimed to assess the use of PMA coupled with RT-qPCR to distinguish between infectious and inactive CGMMV viruses in seeds and plant tissues. The objectives were to: (i) design specific primers for CGMMV based on coat protein (*cp*) gene sequences; (ii) optimize the PMA concentration and light exposure time; and (iii) apply the established PMA-RT-qPCR assay to detect infectious viruses in naturally contaminated watermelon leaves, fruits and seeds.

## 2. Materials and Methods

### 2.1. Plant Materials and RNA Extraction

CGMMV-infected watermelon leaves showing typical symptoms of chlorotic mottling were collected from Sanya City in Hainan Province, China, in the growing season of 2020. Nontarget viral isolates, including *Watermelon mosaic virus* (WMV), *Cucumber mosaic virus* (CMV), *Melon mosaic virus* (MMV), *Tobacco ring spot virus* (TRSV), *Cucurbit chlorotic yellows virus* (CCYV), *Zucchini yellow mosaic virus* (ZYMV), *Squash mosaic virus* (SqMV), *Melon yellow spot virus* (MYSV), *Melon necrotic spot virus* (MNSV), *Prunus necrotic ringspot virus* (PNRSV), *Papaya ringspot virus* (PRSV), *Tobacco mosaic virus* (TMV) and *Potato Y virus* (PVY), were provided by the Institute of Vegetables and Flowers, Chinese Academy of Agricultural Sciences (Table 1). All of the virus isolates used in this study were detected and confirmed previously by RT-PCR and stored at −80 °C.

Total RNA was extracted by using an RNA Easy Fast Plant Tissue Kit (TianGen Biotech Co., Ltd., Beijing, China) following the manufacturer’s instructions. Then, 1% agar gel electrophoresis was performed to determine RNA integrity. RNA concentration and purity were measured by using a SmartSpec Plus spectrophotometer (Bio-Rad Laboratories Inc., Hercules, CA, USA). RNA samples with A260/A280 ratios within the range of 1.9–2.1 were used as templates for RT-qPCR. The RNA samples were stored at −80 °C.

### 2.2. Primer Design and RT-PCR Amplificati on

A primer pair cp-3-1F (5′-CCGCTAGGGCTGAGATAG-3′) and cp-3-1R (5′-CTTAGAGGTGGTAGCCTCTGA-3′) was designed for specific amplification of CGMMV RNA based on sequence alignments of the *cp* gene (GenBank: KY977430.1). The virus isolates used for studying the primer specificity in this study are listed in Table 1.

For cDNA synthesis, reverse transcription was performed from 2 μg of total RNA using a FastKing RT Kit (Tiangen, KR116, Beijing, China). After reverse transcription, 1 μL of cDNA was used as the PCR template in a total volume of 25 μL, containing 12.5 μL 2 × Taq PCR Master Mix (MT201, Biomed, Beijing, China) and 0.5 μL of each primer. Amplification was performed in a PCR amplifier (C1000, Bio-Rad Laboratories Inc., Hercules, CA, USA) with an initial denaturation step at 94 °C for 4 min, followed by 34 cycles at 94 °C for 30 s, 60 °C for 30 s, 72 °C for 45 s and a final extension of 72 °C for 10 min. The amplification products of CGMMV isolates were resequenced, and the identity of the sequences was checked against the NCBI database http://blast.ncbi.nlm.nih.gov/Blast.cgi (accessed on 11 May 2020).

The confirmed CGMMV virus products were purified, connected to the M5 HiPer pTOPO-TA vector (Mei5bio, Beijing, China), transformed into DH5α cells (9057, Takara, Dalian, China), inoculated onto Luria-Bertani (LB) plates (10.0 g of tryptone, 5.0 g of yeast extract, 10.0 g of NaCl and 15.0 g of agar per liter; pH 7.0) containing 50 μL/mL ampicillin and then incubated overnight at 37 °C to obtain the plasmid containing the CGMMV *cp* gene. Then, single positive plaques were picked and sequenced for comparison with the known CGMMV *cp* gene sequences. The concentration of the plasmid was measured using an ultra-microspectrophotometer (NanoDrop 2000, Thermo Fisher, Waltham, MA, USA). The copy number of plasmid was calculated according to Fronhoffs et al. (2002) [42].

### 2.3. RT-qPCR Conditions and Quantification Standards

A standard curve for the quantification of CGMMV was generated by analyzing a 10-fold dilution series of plasmid with known quantities from 1.57 × 10^8^ to 1.57 × 10 copies·μL^−1^, which were prepared in sterile distilled water. RT-qPCR was conducted in a 20 μL reaction volume containing 1 μL plasmid DNA, 10 μL ChamQ Universal SYBR qPCR Master Mix (Q711, Vazyme, Nanjing, China), and 0.4 μL of each primer cp-3-1F/cp-3-1R. Amplification was performed in the real-time PCR system (ABI 7500, Thermo Fisher, Waltham, MA, USA) under cyclic conditions as follows: 95 °C for 30 s, followed by 40 cycles at 95 °C for 10 s and 60 °C for 30 s. Cycle threshold (Ct) values were calculated automatically by ABI 7500 software (Thermo Fisher, Waltham, MA, USA). Each dilution was analyzed in ten replicates. The standard deviation (SD) of the Ct values was determined based on ten replicates, and the coefficient of variation (CV) was calculated with the SD and the mean values of Ct (CV = 100 × SD/mean). The detection limit of RT-qPCR was specified as the lowest concentration at which replicates showed CV ≤ 35% at the calculated concentration [43,44].

### 2.4. Preparation of Virus Suspensions

The CGMMV virus suspensions were prepared according to the method described by Jones et al. (1986) and Zhang et al. (2016) [45,46]. Briefly, 20 g of CGMMV positive tissues was ground with liquid nitrogen, homogenized in 0.1 M phosphate buffer at pH 7.0 with an equal volume of n-butanol/chloroform (1:1, *v*/*v*) for 2–3 min at room temperature in a vortex oscillator. The aqueous layer containing the virus was separated from the organic phase by centrifugation at 900 g. The virus was then subjected to one or two more cycles of differential centrifugation in 0.1 M phosphate at pH 7.0, taking up the pellets in water. The obtained virus suspensions of CGMMV were then divided and prepared into two groups: infectious virus (not treated) and inactive virus (heat-killed in a hot bath at 100 °C for 20 min).

### 2.5. Optimization of PMA Concentration

The appropriate concentration of PMA would be an important factor to effectively discriminate between infectious virus and inactive virus, which should inhibit the amplification of inactive virus and have no effect on the amplification of infectious virus. PMA (Biotium, San Francisco Bay Area, CA, USA) was dissolved in 20% DMSO to create a stock concentration of 20 mmol·L^−1^, which was stored at −20 °C in the dark. Briefly, 200 μL of either infectious or inactive virus suspension of CGMMV was treated with PMA at different final concentrations of 0, 10, 40, 80, 120, 160 and 200 μmol·L^−1^ in Eppendorf tubes. The tubes were first incubated for 30 min at room temperature in the dark, which allowed PMA to penetrate the inactive virus and bind to the RNA. The tubes were then placed in an ice bath with lids removed and exposed to light from a 50 W LED lamp for 15 min at a distance of 15 cm from the sample tube to activate PMA in inactive virus and to photolyze free PMA in solution. After PMA treatment, CGMMV RNA was extracted as described above for amplification by RT-qPCR. The optimal PMA concentration was determined by calculating and comparing the dCt and ddCt of PMA-treated or PMA-untreated samples of infectious or inactive viruses, with ddCt = dCt (inactive) − dCt (infectious), dCt = Ct (+PMA) − Ct (−PMA) [47]. Each sample was treated and assayed by RT-qPCR in triplicate to ensure the reproducibility of the result.

### 2.6. Detection of the Defined Ratio of Mixed Virus Suspension

To evaluate the efficacy of the PMA-RT-qPCR assay to selectively detect the infectious CGMMV virus in the presence of inactive CGMMV virus, PMA treatments were performed in predefined ratios of CGMMV virus suspensions (0%, 10%, 30%, 50%, 70%, 90% and 100% infectious CGMMV virus). Each sample, containing different numbers of infectious and inactive viruses, was divided into two aliquots and assessed in two different ways: (i) PMA pretreatment followed by RT-qPCR (three parallel qPCRs per replicate) and (ii) RT-qPCR (three per replicate), respectively. Three replicates were performed for each of the two different assays.

### 2.7. Sampling of Naturally Infected Watermelon Tissues

A total of 13 leaf, 3 fruit and 6 seedlot samples were collected from CGMMV-infected fields during 2020–2021 (Table 2). The leaf and fruit samples showing suspected symptoms were collected from Ningbo city of Zhejiang Province (4 leaf and 2 fruit samples), Sanya city of Hainan Province (4 leaf and 1 fruit samples), Yingkou city of Liaoning Province (3 leaf samples) and Zhongwei city of Ningxia Hui Autonomous Region (2 leaf samples), China. The seedlots were collected from Ningbo city of Zhejiang Province (3 seedlots), Sanya city of Hainan Province (2 seedlots) and Zhongwei city of Ningxia Hui Autonomous Region of China (1 seedlot). These seedlots, which ranged from 50 to 100 g, were kindly donated by local farmers shortly after harvest and had not been commercially cleaned.

### 2.8. Detection of CGMMV from Naturally Infected Watermelon Tissues

All naturally infected watermelon leaf, fruit and seedlot samples as well as controls were assessed for the quantities of total or infectious CGMMV virus by RT-qPCR and PMA-RT-qPCR, respectively. In addition, the infection rates of seedlot samples were detected by bioassays.

For RT-qPCR and PMA-RT-qPCR, the protocol for preparing CGMMV virus suspensions from infected contaminated leaf, fruit and seedlot samples was based on the methods of Jones et al. (1986) and Zhang et al. (2016) as described previously [45,46]. The virus suspension was then divided into two aliquots and assessed by (i) RT-qPCR and (ii) 120 μmol·L^−1^ PMA pretreatment followed by RT-qPCR, respectively. Each sample was replicated three times, and each replicate was analyzed by three parallel RT-qPCRs (for a total of nine RT-qPCR amplifications per original sample). The average Ct value for each sample was calculated based on the analysis of nine RT-qPCR amplifications.

For bioassays, 60 seeds were selected randomly from each of 6 seedlots and sown in 20 cm diameter plastic pots filled with sterile substrate. The pots were kept in a greenhouse at 18–20 °C (night)/26–30 °C (day) with 80–100% relative humidity under natural daylight conditions. Four weeks later, symptoms of leaf mottling and mosaic were checked, and the disease incidence (DI) was examined according to the following equation: DI = no. of diseased plants/total no. of plants × 100%. The bioassay was repeated three times.

### 2.9. Statistical Analysis

All statistical analyses were performed using SPSS 17.0 software (IBM, Armonk, New York, NY, USA). A one-way analysis of variance (ANOVA), with a significance level of *p* < 0.05, was carried out to determine significant differences between the means. Ct and DI values are expressed as the means ± SD.

## 3. Results

### 3.1. Specificity of Primers

**Figure 1:**
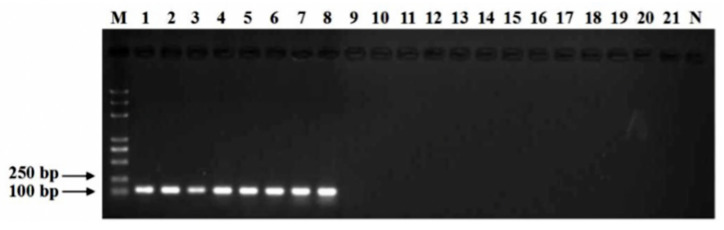
Specific detection of the *cp* gene in *Cucumber green mottle mosaic virus* (CGMMV) by the primer pair cp3-1F/cp3-1R. Lane M, BM 5000 bp DNA marker; Lanes 1 to 8, CGMMV (TG20091901, TG20091902, TG20081101, TG20081102, TG21061411, TG21061412, TG21121234 and TG21121235); Lane 9, *Watermelon mosaic virus* (HG16092601); Lane 10, *Cucumber mosaic virus* (TG18041704); Lane 11, *Melon mosaic virus* (TG19011201); Lane 12, *Tobacco ring spot virus* (HG18092107); Lane 13, *Cucurbit chlorotic yellows virus* (HG18111501); Lane 14, *Zucchini yellow mosaic virus* (NG17071124); Lane 15, *Squash mosaic virus* (SQ20041046); Lane 16, *Melon yellow spot virus* (MY20075501); Lane 17, *Melon necrotic spot virus* (MN21060102); Lane 18, *Prunus necrotic ringspot virus* (PNR20070108); Lane 19, *Papaya ringspot virus* (PR20040101); Lane 20, *Tobacco mosaic virus* (FQ18062901); Lane 21, *Potato Y virus* (MLS18080202); N, negative control.

Specificity analyses using the primer pair cp3-1F/cp3-1R showed positive results for all eight isolates of CGMMV virus, while no amplification products were obtained from the other related virus isolates of WMV, CMV, MMV, TRSV, CCYV, ZYMV, SqMV, MYSV, MNSV, PNRSV, PRSV, TMV and PVY (Figure 1). The amplification of purified CGMMV cDNA using the primers cp3-1F/cp3-1R produced a PCR product 119 bp in size on a 1% agar gel. A sequence analysis of the amplified products showed 100% similarity with the sequences of CGMMV-NMG isolates (GenBank: KY977430.1), thereby verifying the identity of the amplified fragment.

### 3.2. Standard Curve and Sensitivity of RT-qPCR

**Figure 2:**
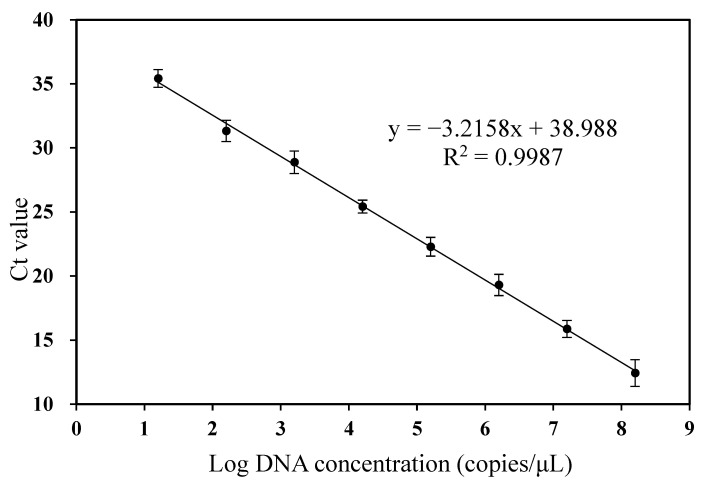
Standard curve of quantitative real-time reverse-transcription PCR (RT-qPCR) generated using a 10-fold serial dilution of CGMMV plasmid ranging from 1.57 × 10^8^ to 1.57 × 10 copies·μL^−1^. Log values of the copy number were plotted against the corresponding threshold cycle (Ct) values obtained by RT-qPCR, and the linear regression equation and correlation coefficient (R^2^) are displayed in the graph. Error bars represent the standard deviation from three replicate reactions.

A quantification standard curve was constructed by a dilution series of CGMMV plasmid ranging from 1.57 × 10^8^ to 1.57 × 10 copies·μL^−1^. A good linear relationship between the log of the concentration of CGMMV plasmid and the corresponding Ct values was obtained (y = −3.2158x + 38.827, R^2^ = 0.9987) (Figure 2). The amplification efficiency was 100%. Thus, the primer pair cp3-1F/cp3-1R was specific and efficient and could be used for the accurate quantification of CGMMV. The dilution of pure CGMMV plasmid at a concentration of 1.57 × 10^2^ copies·μL^−1^ per reaction produced good reproducibility (CV < 35), and the corresponding Ct value was 31.33. A lower concentration of CGMMV plasmid 1.57 × 10 copies·μL^−1^ per reaction resulted in poor reproducibility (CV > 35), giving a Ct value of 35.43. Hence, the detection limit of the RT-qPCR assay was 1.57 × 10^2^ copies·μL^−1^ of CGMMV plasmid.

### 3.3. Optimization of the PMA-RT-qPCR System

In the PMA-RT-qPCR assay, an adequate concentration of PMA should be added to inhibit the amplification of target RNA from inactive CGMMV virus and quantify target RNA from infectious CGMMV virus. Hence, the efficiency and toxicity of a range of PMA concentrations were tested with infectious and inactive virus suspensions of CGMMV.

For inactive CGMMV virus, when the PMA concentration increased from 0 to 120 μmol·L^−1^, the Ct values increased significantly (*p* < 0.05), which suggested that the inhibition of amplification of the RNA from inactive virus significantly increased with increasing PMA concentration. When the PMA concentration was equal to or greater than 120 μmol·L^−1^, the Ct values were greater than 31 and remained constant, implying that the application of RNA from inactive CGMMV was completely prevented (Table 2). Therefore, 120 μmol·L^−1^ was considered the minimum optimal PMA concentration that inhibited the RT-qPCR amplification of RNA from inactive CGMMV virus suspensions.

For infectious CGMMV virus, as the PMA concentration increased from 0 to 120 μmol·L^−1^, the Ct values did not change significantly (*p* > 0.05), which suggested that PMA had a similar performance at these concentrations. However, when the PMA concentration increased from 120 to 200 μmol·L^−1^, the Ct values increased significantly (*p* < 0.05), indicating that high PMA concentrations (> 120 μmol·L^−1^) exerted toxicity toward the infectious CGMMV virus (Table 2). The toxicity could be explained by the infiltration of infectious virus by excessive PMA.

**Figure 3:**
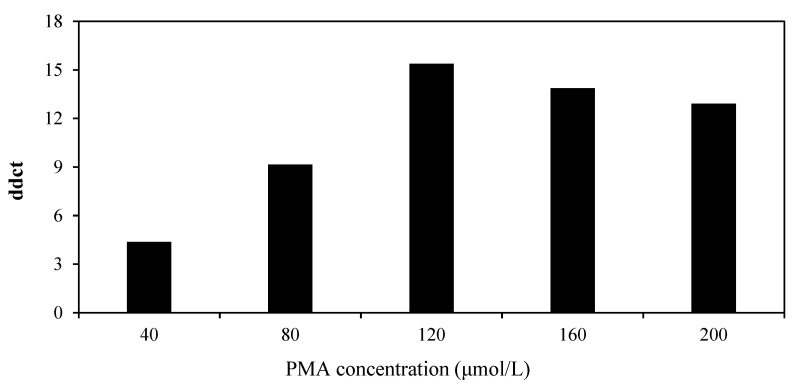
Effect of PMA concentration on the amplification of RNA from infectious and inactive CGMMV. Infectious and inactive CGMMV virus suspensions were exposed to different concentrations of PMA (0, 40, 80, 120, 160 and 200 μmol·L^−1^). ddCt = dCt (inactive) − dCt (infectious); dCt = Ct (+PMA) − Ct (−PMA).

In addition, as the concentration of PMA increased, the ddCt value initially increased and then decreased, reaching a maximum of 15.39 at a concentration of 120 μmol·L^−1^ (Table 2, Figure 3). Thus, to completely inhibit the amplification of RNA from inactive virus and have no significant effect on infectious virus, 120 μmol·L^−1^ PMA was the most suitable final concentration.

### 3.4. Verification and Application of the PMA System

**Figure 4:**
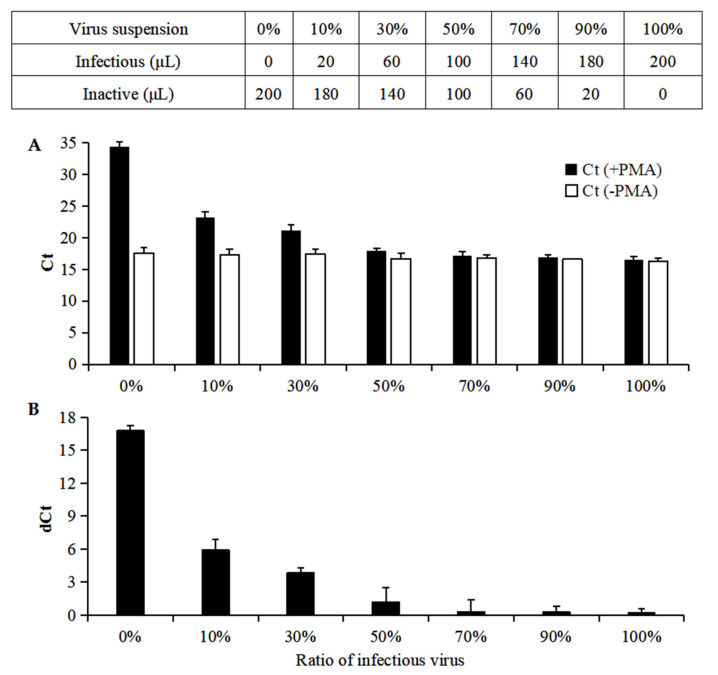
Effectiveness of PMA-assisted RT-qPCR in detecting infectious CGMMV from viral mixtures with defined ratios. Infectious and heat-inactivated CGMMV viruses were mixed in predefined ratios (0%, 10%, 30%, 50%, 70%, 90% and 100%), and the concentrations of total or infectious CGMMV viruses were determined by RT-qPCR and PMA-RT-qPCR assays. (**A**) Ct values and (**B**) dCt values were obtained by RT-qPCR and PMA-RT-qPCR assays. dCt = Ct (+PMA) − Ct (−PMA).

Mixtures containing predefined ratios of infectious and inactive CGMMV virus suspensions (0%, 10%, 30%, 50%, 70%, 90% and 100% of infectious CGMMV virus) were assessed by RT-qPCR and PMA-RT-qPCR assays. For RT-qPCR, when the ratios of infectious virus increased from 0% to 100%, the Ct values remained nearly unchanged in the range of 16.31 ± 0.42 to 17.60 ± 0.80 (*p* > 0.05). For PMA-RT-qPCR, as the ratio of infectious virus increased from 0% to 100%, the Ct values decreased gradually from 34.38 ± 0.81 to 16.54 ± 0.45 (*p* < 0.05) (Figure 4A). The dCt, which was calculated by Ct (+PMA) − Ct (−PMA), decreased gradually with an increase in the ratio of infectious viruses (Figure 4B). dCt values > 16 were observed for 100% inactive virus, and dCt values of 0.23 were obtained for 100% infectious virus. These results proved that the PMA-assisted RT-qPCR assay was a suitable tool for selectively quantifying infectious CGMMV viruses.

### 3.5. Application of PMA-RT-qPCR to Naturally Infected Watermelon Tissues

All naturally infected leaf, fruit and seedlot samples of watermelon were tested for the presence of total and infectious CGMMV virus using RT-qPCR and PMA-RT-qPCR, respectively. CGMMV virus could be reliably and unequivocally quantified in 13 out of the 22 watermelon tissue samples both by RT-qPCR and by PMA-RT-qPCR, with an infection rate of 59.09%. Nevertheless, the quantities of virus detected by RT-qPCR were higher than those detected by PMA-RT-qPCR. The Ct values detected by RT-qPCR ranged from 18.93 to 29.31, and the corresponding concentration of total CGMMV virus in infected tissues was 9.09 × 10^2^~1.54 × 10^6^ copies per gram. The Ct value detected by PMA-RT-qPCR was higher than that detected by RT-qPCR, which was in the range of 20.48~31.37, and the corresponding concentration of active CGMMV virus was 2.08 × 10^2^~5.08 × 10^5^ copies per gram (Table 3).

The highest levels of infectious CGMMV virus were detected in leaf samples originating from Ningbo City, Zhejiang Province (sample 1) and Sanya City, Hainan Province (sample 6), which yielded Ct values of 21.37 ± 0.82 and 20.48 ± 0.47 by PMA-RT-qPCR, equivalent to 2.68 × 10^5^ and 5.08 × 10^5^ copies per gram of leaves, respectively. Leaf and fruit samples collected from Ningbo City, Zhejiang Province (samples 2, 4 and 9), Yingkou City, Liaoning Province (sample 5) and Zhongwei City, Ningxia Hui Autonomous Region (Samples 8 and 11) gave Ct values ranging from 23.27 ± 0.83 to 28.01 ± 1.40, corresponding to CGMMV copy numbers of 2.30 × 10^3^ to 6.89 × 10^4^ copies per gram. The quantities of infectious CGMMV virus were fairly low in leaf and fruit samples originating from Ningbo City, Zhejiang Province (samples 3 and 10) and Sanya City, Hainan Province (sample 7), with 5.24 × 10^2^ to 7.04 × 10^2^ copies per gram (Table 3).

Infectious CGMMV virus was also found on seedlots harvested from Ningbo City, Zhejiang Province (Samples 13 and 14), which gave Ct values of 28.50 ± 0.46 and 31.37 ± 0.42 by PMA-RT-qPCR, carrying 1.63 × 10^3^ and 2.08 × 10^2^ copies per gram of seeds, respectively (Table 3). In the bioassays, typical symptoms of CGMMV disease developed on the two PMA-RT-qPCR-positive seedlot samples (Table 3), with DI values of 31.11 ± 0.11 (sample 5) and 21.67 ± 0.07, respectively (sample 6). No symptoms were observed in the other four PMA-RT-qPCR-negative watermelon seedlot samples.

## 4. Discussion

In recent years, CGMMV has emerged as a devastating virus of cucurbit crops that can cause substantial economic losses worldwide [16,17,48]. The seed-borne and seedling transmission of CGMMV is one of the principal modes for disease dispersal [21]. Hence, the use of virus-free seeds and seedlings is of great importance for disease prevention and control, which relies on the availability of sensitive specific methods that can be used for the rapid and accurate detection of CGMMV.

Several molecular methods, e.g., RT-PCR, IC-RT-PCR, RT-LAMP and RT-qPCR, have been developed for the detection of CGMMV [24,25,26,27,28]. However, in these assays, RNA derived from dead CGMMV virus that does not have the ability to infect also showed a positive reaction, which may lead to an overestimation of the disease damage. PMA can covalently react with the RNA of inactive viruses upon light irradiation, and PMA–RNA conjugates can block PCR amplification [34,38,49]. The PMA–RT-qPCR system for CGMMV established in this study is the first attempt to identify infectious and inactive plant virus samples, which is of great significance for the prevention of subsequent plant virus diseases.

In the present study, the RT-qPCR specific primers cp3-1F and cp3-1R were developed for the specific detection of CGMMV based on the *cp* gene, which produced strong positive results for all eight CGMMV isolates, while no signals were obtained from the other 13 virus isolates tested. In the RT-qPCR assay, when the concentration of CGMMV plasmid DNA was less than 1.57 × 10^2^ copies·μL^−1^, the Ct values were greater than 31, which indicated that the detection limit of the RT-qPCR assay was 10^2^ copies·μL^−1^ of CGMMV plasmid.

The PMA concentration is the main key factor that affects the efficiency of the PMA-RT-qPCR assay to discriminate between infectious and inactive viruses. In this study, the concentration of PMA was optimized to distinguish between infectious and inactive CGMMV viruses. The optimal PMA concentration was 120 μmol·L^−1^. PMA-RT-qPCR has been applied to detect active human and environmental viruses in several previous studies [34,35,36,37,38,39,40,50]. Among these studies, there is little consensus on optimal PMA concentrations, which range from 5 to 100 μmol·L^−1^. For the detection of infectious SARS-CoV-2, the optimal PMA concentration was 50 μmol·L^−1^ coupled with 0.005% SDS [40]. To detect infectious enterovirus and norovirus, e.g., poliovirus 1, MNV-1 and Norwalk virus, the optimal concentration of PMA was 348 μmol·L^−1^ [37]. Although dark incubation time and light exposure time may affect the efficiency of the PMA-RT-qPCR assay, recent studies have shown that these conditions have little effect [37,39,41,50]. In this study, the dark incubation time and light exposure time were set at 15 min and 30 min, respectively.

The consistency and reliability of the PMA-RT-qPCR assay were confirmed. When the virus samples were detected directly by RT-qPCR, no significant differences were found among the different defined ratios of virus suspensions (0%, 10%, 30%, 50%, 70%, 90% and 100% infectious CGMMV virus). However, after PMA pretreatment, the Ct values decreased significantly as the proportion of infectious CGMMV virus increased from 0% to 100%. Correspondingly, the dCt values also increased gradually. However, due to the difficulty of plant virus culture, virus suspensions prepared from infected tissues may contain both infectious and noninfectious virus; therefore, the maximum value of dCt could not be detected based on fully viable and completely inactive CGMMV virus samples. To minimize the unwanted interference, infected tissues were freshly collected, and virus suspensions were prepared immediately. The method established in this study was feasible for the mixed detection of infectious and inactive virus samples with a defined ratio. In addition, it has been shown that surfactants can optimize RT-qPCR results by weakening the viral protein envelope [51,52,53], making it easier for PMA to penetrate the virus and bind to RNA [40]. Future studies may optimize PMA-RT-qPCR targeting CGMMV using surfactant-assisted methods.

The PMA-RT-qPCR assay was further employed for the selective detection of infectious CGMMV viruses in naturally infected watermelon leaves, fruits and seedlots. In total, 22 watermelon tissue samples suspected to be infected with CGMMV were collected from different parts of China and detected by PMA-RT-qPCR. Quantifiable CGMMV viruses were found in eight leaf samples, three fruit samples and two seedlot samples, indicating that infectious CGMMV viruses could be detected from naturally infected leaves, fruits and seeds.

In conclusion, the PMA-RT-qPCR assay developed in the current study was an effective, sensitive and reliable assay to quantitatively detect infectious CGMMV virus from leaves, fruits and seedlots of cucurbit crops. Therefore, the PMA-RT-qPCR assay can serve as a useful tool to be used for monitoring and early warning of CGMMV virus for commercial cucurbit producers.

## Figures and Tables

**Table 1:** Representative virus isolates used for primer specificity test.

Viruses	Isolate Code	Host	Tissues	Geographic Origin	PCR ^a^
*Cucumber green mottle mosaic virus* (CGMMV)	TG20091901	Watermelon	Leaves	Zhejiang Province	+
*Cucumber green mottle mosaic virus* (CGMMV)	TG20091902	Watermelon	Leaves	Zhejiang Province	+
*Cucumber green mottle mosaic virus* (CGMMV)	TG20081101	Watermelon	Leaves	Hainan Province	+
*Cucumber green mottle mosaic virus* (CGMMV)	TG20081102	Watermelon	Leaves	Hainan Province	+
*Cucumber green mottle mosaic virus* (CGMMV)	TG21061411	Watermelon	Leaves	Liaoning Province	+
*Cucumber green mottle mosaic virus* (CGMMV)	TG21061412	Watermelon	Leaves	Liaoning Province	+
*Cucumber green mottle mosaic virus* (CGMMV)	TG21121234	Watermelon	Leaves	Ningxia Hui Autonomous Region	+
*Cucumber green mottle mosaic virus* (CGMMV)	TG21121235	Watermelon	Leaves	Ningxia Hui Autonomous Region	+
*Watermelon mosaic virus* (WMV)	HG16092601	Cucumber	Leaves	Shandong province	−
*Cucumber mosaic virus* (CMV)	TG18041704	Melon	Leaves	Shandong province	−
*Melon mosaic virus* (MMV)	TG19011201	Melon	Leaves	Shandong province	−
*Tobacco ringspot virus* (TRSV)	HG18092107	Cucumber	Leaves	Shandong Province	−
*Cucurbit chlorotic yellows virus* (CCYV)	HG18111501	Cucumber	Leaves	Henan Province	−
*Zucchini yellow mosaic virus* (ZYMV)	NG17071124	Pumpkin	Leaves	Beijing	−
*Squash mosaic virus* (SqMV)	SQ20041046	Melon	Leaves	Beijing	−
*Melon yellow spot virus* (MYSV)	MY20075501	Melon	Leaves	Guangxi Zhuang Autonomous Region	−
*Melon necrotic spot virus* (MNSV)	MN21060102	Melon	Leaves	Shandong Province	−
*Prunus necrotic ringspot virus* (PNRSV)	PNR20070108	Melon	Leaves	Shanxi Province	−
*Papaya ringspot virus* (PRSV)	PR20040101	Papaya	Leaves	Guangdong Province	−
*Tobacco mosaic virus* (TMV)	FQ18062901	Tomato	Leaves	Inner Mongolia Autonomous Region	−
*Potato Y virus* (PVY)	MLS18080202	Potato	Leaves	Hebei Province	−

^a^ PCR results are scored as + for positive reaction and − for negative reaction.

**Table 2:** Effect of PMA concentration on the amplification of RNA from infectious and inactive CGMMV.

PMA Concentration (μmol·L^−1^)	Ct (Infectious Virus)	Ct (Inactive Virus)	ddCt ^1^
0	16.01 ± 0.36 c ^2^	16.38 ± 0.33 d	-
40	16.11 ± 0.26 c	20.40 ± 0.40 c	4.37
80	16.33 ± 0.13 c	25.41 ± 0.39 b	9.15
120	16.12 ± 0.44 c	30.43 ± 0.41 a	15.39
160	17.66 ± 0.14 b	31.46 ± 0.39 a	13.87
200	19.40 ± 0.25 a	32.24 ± 0.41 a	12.91

^1^ ddCt = dCt (inactive) − dCt (infectious); dCt (inactive) = Ct (inactive with PMA) − Ct (inactive without PMA); dCt (infectious) = Ct (infectious with PMA) − Ct (infectious without PMA). ^2^ ‘±’ means standard deviation. Means followed by different letters are significantly different (*p* < 0.05).

**Table 3:** Detection of total and infectious CGMMV in naturally infected tissues by RT-qPCR, PMA-RT-qPCR and seed bioassay ^1^.

Sample No.	Collection Sites	Sampling Year	Tissues	RT-qPCR		PMA–RT-qPCR		Bioassay ^1^ DI (%) ^2^
				Ct Value	Total Virus (Copies/g)	Ct Value	Infectious Virus (Copies/g)	
1	Ningbo, Zhejiang	2021	Leaf	19.97 ± 0.33	7.30 × 10^5^	21.37 ± 0.82	2.68 × 10^5^	/
2	Ningbo, Zhejiang	2020	Leaf	20.82 ± 0.19	3.99 × 10^5^	23.27 ± 0.83	6.89 × 10^4^	/
3	Ningbo, Zhejiang	2021	Leaf	27.01 ± 0.34	4.72 × 10^3^	29.67 ± 0.80	7.04 × 10^2^	/
4	Ningbo, Zhejiang	2020	Leaf	23.52 ± 0.30	5.75 × 10^4^	28.01 ± 1.40	2.30 × 10^3^	/
5	Yingkou, Liaoning	2020	Leaf	24.41 ± 1.10	3.05 × 10^4^	27.75 ± 0.49	2.78 × 10^3^	/
6	Sanya, Hainan	2021	Leaf	18.93 ± 0.66	1.54 × 10^6^	20.48 ± 0.47	5.08 × 10^5^	/
7	Sanya, Hainan	2020	Leaf	29.27 ± 0.31	9.37 × 10^2^	30.08 ± 0.43	5.24 × 10^2^	/
8	Zhongwei, Ningxia	2020	Leaf	20.85 ± 1.34	3.90 × 10^5^	25.44 ± 2.37	1.46 × 10^4^	/
9	Ningbo, Zhejiang	2021	Fruit	22.99 ± 0.72	8.41 × 10^4^	24.99 ± 1.79	2.00 × 10^4^	/
10	Ningbo, Zhejiang	2021	Fruit	28.15 ± 0.66	2.09 × 10^3^	29.51 ± 0.41	7.89 × 10^2^	/
11	Zhongwei, Ningxia	2020	Fruit	23.01 ± 1.12	8.27 × 10^4^	27.29 ± 1.61	3.87 × 10^3^	/
12	Ningbo, Zhejiang	2021	Seed	29.31 ± 0.18	9.09 × 10^2^	31.37 ± 0.42	2.08 × 10^2^	21.67 ± 0.07
13	Ningbo, Zhejiang	2021	Seed	24.12 ± 0.48	3.74 × 10^4^	28.50 ± 0.46	1.63 × 10^3^	31.11 ± 0.11
The other 9 samples				>32	0	>32	0	/

^1^ For seed bioassays, 60 seeds were selected randomly from each of 6 seedlots and sown in 20 cm diameter plastic pots filled with sterile substrate. The pots were kept in a greenhouse at 18–20 °C (night)/26–30 °C (day) with 80–100% relative humidity under natural daylight conditions. ^2^ Disease incidence (DI) was calculated by the formula DI = (number of diseased plants/total number of plants) × 100%, and the DI values represent the means ± SDs of three replicates.

## Data Availability

The data presented in this study are available upon request from the corresponding author.
